# Medical Correspondents

**Published:** 1921-10

**Authors:** 


*To the Editor of* THE HOSPITAL AND HEALTH REVIEW.

### "Medical Correspondents."

*To the Editor of* THE HOSPITAL AND HEALTH REVIEW.

SIR,—I have noticed in recent years a growing disposition on the part of the public to acquire medical knowledge, what time they are thrilled or depressed by glowing headlines in their morning paper. And in searching for the channel through which the jaded city man acquires disconcerting facility in manipulating pathological terminology in the consulting-room (and in the train) one is arrested by the likelihood of this being provided by ingenious publicists, who lay out bodily and social evils in contiguous columns of the Press. Here is an example of what can be done by taking advantage of temporary keen receptivity of the human mind when there is no urgent necessity for it to function at the full. We are all troubled by the haunting memory of some trivial advertisement in the Tube, or 'bus, which, scarcely glanced at, yet lingers to the exclusion of more valuable matter, and in taking advantage of unconscious cerebration the medical correspondent is just as much an opportunist as the mother who wraps up a pill in a spoonful of jam-roll for her unsuspecting offspring.

So that the aim should be to give what is best, and to give it at the right moment. To-day there

**CORRESPONDENCE—(*cont.*).**

is great need for popular opinion to have a firm grounding in those principles which make successful practice in the public-health services—to be told what difficulties the various organisations encounter. Side by side with an announcement that the housing scheme has been dropped, or a strike declared, or a small-pox outbreak discovered, there is the proper space for an incisive article on the results of over-crowding, the consequences of under-nourishment, the dangers to the unvaccinated. The medical contributor will be called upon to use a nice restraint and a fine discrimination, avoiding undue pessimism or optimism on the one hand, curbing a righteous wrath, or foregoing partisan spirit on the other. The public looks to him who stands for authority in things medical to tell them of what is best in his sphere, whilst we who are doctors expect that in the doing of it our dignity shall be upheld, our true interests furthered, out just claims recognised, and our successes rightly valued.

The good which should accrue to the profession must be impersonal, collective—not tainted with the suspicion of an ulterior motive. A sound practical public-health man on the staff of every great daily newspaper would confer a blessing on the country, which it is hard immediately to realise. Undoubtedly an interest in national sanity heretofore unknown is being evinced by the people, and whilst this interest is awake it should be kept alert lest our natural inclination towards lethargy reasserts itself.

But such stuff as I remember seeing retailed in certain weeklies, or thumb-nail sketches of the tænidæ, defeat the object to which medical correspondents, in my opinion, should strive to attain. The one simply bespeaks lack of confidence in the practitioner, the other reflects a distorted idea of what is good for the reader. Life is harrowing enough without having disquisitions on hob-nail liver before you at breakfast, without having the tactics of the tapeworm dished up in your Sunday paper after the irritating tic-tac of the tape all the week.—I am, yours, &c.,

ANOTHER MEDICAL CORRESPONDENT.

